# MRI/US Fusion Transperineal Versus Transrectral Biopsy of Prostate Cancer: Outcomes and Complication Rates, a Tertiary Medical Center Experience in the Middle East

**DOI:** 10.5152/tud.2022.21248

**Published:** 2022-03-01

**Authors:** Adnan El-Achkar, Nassib Abou Heidar, Muheiddine Labban, Mouhamad Al-Moussawy, Hisham Moukaddem, Rami Nasr, Raja Khauli, Albert El-Hajj, Muhammad Bulbul

**Affiliations:** 1 Division of Urology, Department of Surgery, American University of Beirut Medical Center, Beirut, Lebanon; 2 Department of Diagnostic Radiology, American University of Beirut Medical Center, Beirut, Lebanon

**Keywords:** MRI/US fusion, prostatic neoplasm, transperineal biopsy, transrectal biopsy.

## Abstract

**Objective:**

**: **To report on the outcomes of transperineal versus transrectal magnetic resonance imaging/ultrasound fusion biopsy of the prostate including detection of clinically significant cancer and complications. This is the first and largest series in the Middle East.

**Material and methods:**

Between May 2019 and June 2020, 145 patients with suspicious lesions on magnetic resonance imaging underwent magnetic resonance imaging/ultrasound fusion prostate biopsy at our center. Transperineal biopsy was performed under light sedation, while transrectal biopsy patients had a periprostatic block for anesthesia. Clinically significant cancer was defined as Gleason ≥3 + 4

**Results:**

In all, 98 transperineal biopsies and 47 transrectal magnetic resonance imaging/ultrasound fusion prostate biopsies were done. Patients had similar prebiopsy parameters (transperineal vs. transrectal): median age (64.5 vs. 66 years; *P* = .68), median prostate-specific antigen value (7.5 vs. 7.5; *P* = .42), and median prostate volume (51 vs. 52.5; *P* = .83). Those that underwent transperineal biopsy had fewer average total number of cores compared to transrectal ultrasound-guided biopsy (11 vs. 13; *P* = .025) fewer average number of random cores (3 vs. 6; *P* < .0001), and the detection rate of clinically significant cancer was similar between the groups (44% vs. 48.9%; *P* = .57). No difference in hematuria, retention, and sepsis rate requiring admission (1 vs. 2; *P* = .2) was observed. However, more patients had urinary tract infection in the transrectal ultrasound-guided biopsy group compared to transperineal biopsy group (5 vs. 1; *P* = .006) that were treated with antibiotics on outside basis.

**Conclusion:**

: Magnetic resonance imaging/ultrasound transperineal fusion biopsy has similar detection rate of clinically significant cancer compared to transrectal ultrasound-guided biopsy with less urinary tract infection post biopsy.

## Main Points

- This is the first and the largest series in the Middle East comparing outcomes and complications of magnetic resonance imaging/ultrasound (MRI/US) fusion transrectal versus transperineal biopsy (TP) of the prostate.
- Transrectal ultrasound-guided biopsy (TRUS) and TP MRI/US fusion biopsy of prostate both have similar detection rate of clinically significant cancer.
- Transperineal biopsy is a better tolerated procedure compared to TRUS with less complication post biopsy, especially urinary tract infection.

## Introduction

Transrectal ultrasound-guided (TRUS) biopsy has been the gold standard for prostate biopsy for the last 2 decades.^1^ It is the preferred method used to biopsy the prostate by most urologist in the world, as well as in the United States.^2^ The reported 30-day complication rate is 3.7% and hospital admissions attributed to TRUS is between 0.8% and 6.9% with the majority of complication being due to infections.^3^ The risk of urinary tract infection (UTI) post biopsy ranges between 2% and 6%, and sepsis rate with bacteremia is between 0.1% and 2.2% and has been increasing in recent years due to the excessive use of antibiotics perioperatively.^4,5^ This has facilitated the emergence of antibiotic-resistant bacteria, making a single sepsis episode very costly.^4,6^

With the introduction of magnetic resonance imaging/ultrasound (MRI/US) fusion technology, the adoption of transperineal (TP) biopsy has gained momentum in Europe and elsewhere. Comparative data of TP versus TR showed similar detection rates of clinically significant cancer (CSC) .^7-9^ Low infectious rates post TP biopsy ranges between 0% and 2.4%, while hospital readmissions range between 0% and 1.4%, making it an appealing alternative to TRUS in this era of increasing fluroquinolone-resistant bacterial rectal flora.^10-13^ Growing evidence of negligible sepsis post TP biopsy has pushed many centers in the world to completely abandon the transrectal approach.^14^

Herein, we present the first and largest series comparaing outcomes of MRI/US fusion TRUS versus TP at our institution during the adoption phase, including detection of CSC, complication rates, and risk of upstaging and upgrade post radical prostatectomy. 

## Material and Methods

### Patients and Data Analysis

Between May 2019 and July 2020, retrospective data were collected about 47 patients who underwent MRI/US fusion TR biopsy and 98 patients who underwent MRI/US fusion TP biopsy of prostate. Prior to biopsy, all patients had a 3T multiparametric MRI (mpMRI) of prostate following a rise in prostate-specific antigen (PSA) and/or suspicious digital rectal exam (DRE). All patients underwent biopsy of suspicion lesions identified on MRI. Prostrate image reporting and data system (PIRADS) v2 score was used for classification of lesions on MRI, and Gleason ≥ 3 + 4 was used as a definition for CSC. Following ethical community approval from American University of Beirut (2019-0309) and International Review Board (IRB) approval, clinical data regarding age, prostate size, PSA, medical history, and surgical history were collected retrospectively. Outcomes including detection rate of CSC on biopsy, retention, hematuria, UTI, sepsis, and readmission rate were collected for both groups. In addition, pathology reports of those who underwent radical prostatectomy +/− lymph node dissection were reviewed and rate of upgrade and upstaging were recorded. Medians and interquartile ranges (IQRs) were reported for continuous variables and counts, and percentages were used for categorical variables. We compared demographics, lesion characteristics, and perioperative factors between TRUS and TP using the independent *t*-test for continuous variables and the chi-squared test for categorical variables. The Statistical Package for the Social Sciences^®^ (IBM SPSS Corp.; Armonk, NY, USA) IBM corp^©^ 2017 for MAC OS, version 25 was used to compute the results.

### Biopsy Preparation and Technique

**Figure 1.**
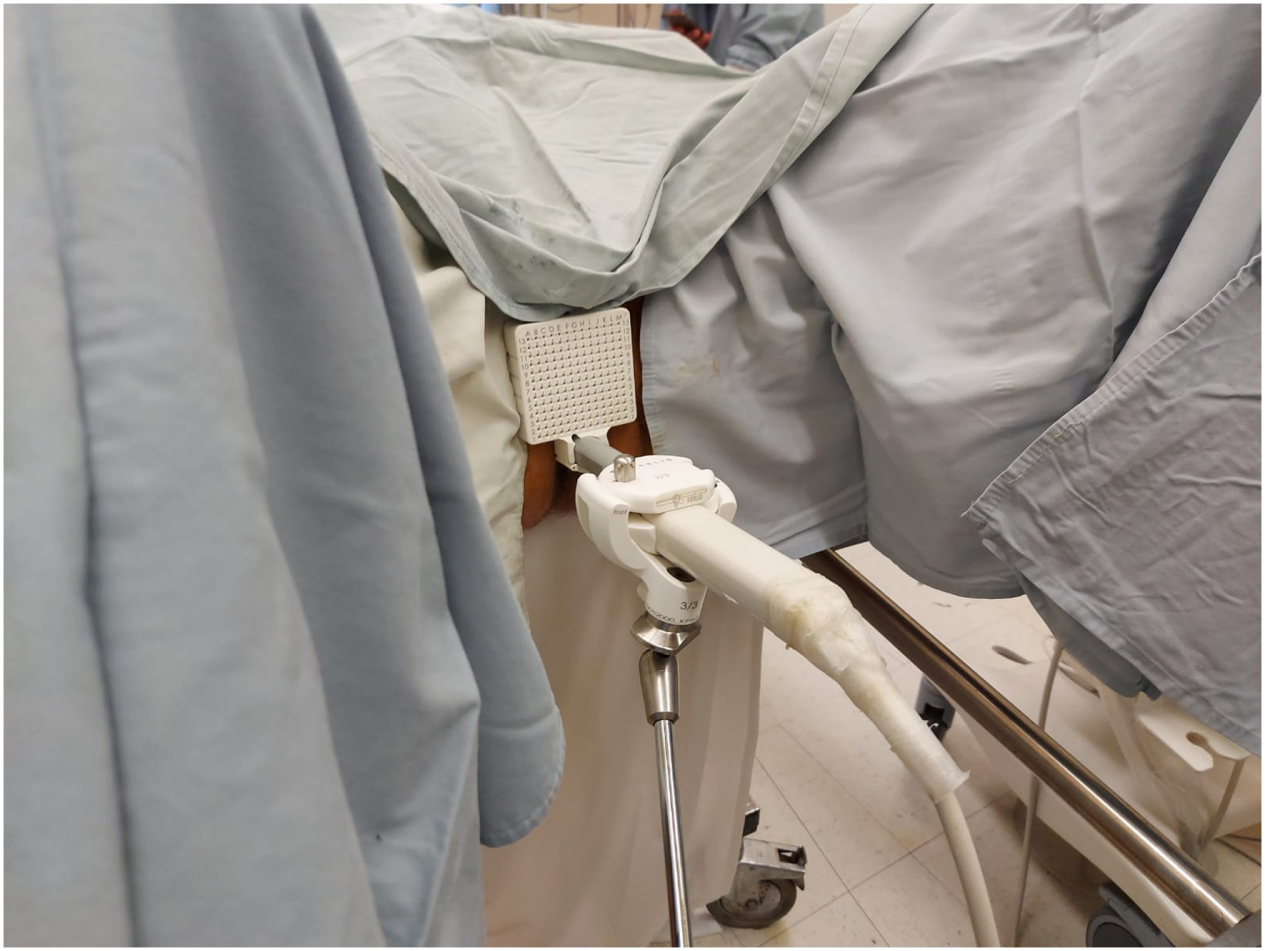
Patient prepped and draped in the lithotomy position. US probe and grid mounted on a stable stepper. US, ultrasound.

Both types of procedure were performed in a dedicated US suite. The team involved a urologist, a nurse, and a specialized uroradiologist. The Koelis Trinity (Koelis, Meylan, Grenoble, France): MRI/US organ-based tracking fusion system is used. All TP biopsies were done under light sedation with the involvement of the anesthesia team. After light sedation, the scrotum is elevated away from the perineum using micropores tape. The perineum is then prepped and draped using betadine and sterile drape in the lithotomy position. The biplanar US probe is advanced into rectum after being clamped on the stepper probe holder. The biopsy perine full grid that is also set on the stepper against the perineum is used to guide the biopsy cores (Figure 1). In the TR approach, biopsy was performed under local anesthesia. The periprostatic block was done using 20 mL of lidocaine 2% without epinephrine. Patients in both groups were required to do fleet enema and take ciprofloxacin 500 mg 1 tab the morning of the procedure. 

**Figure 2.**
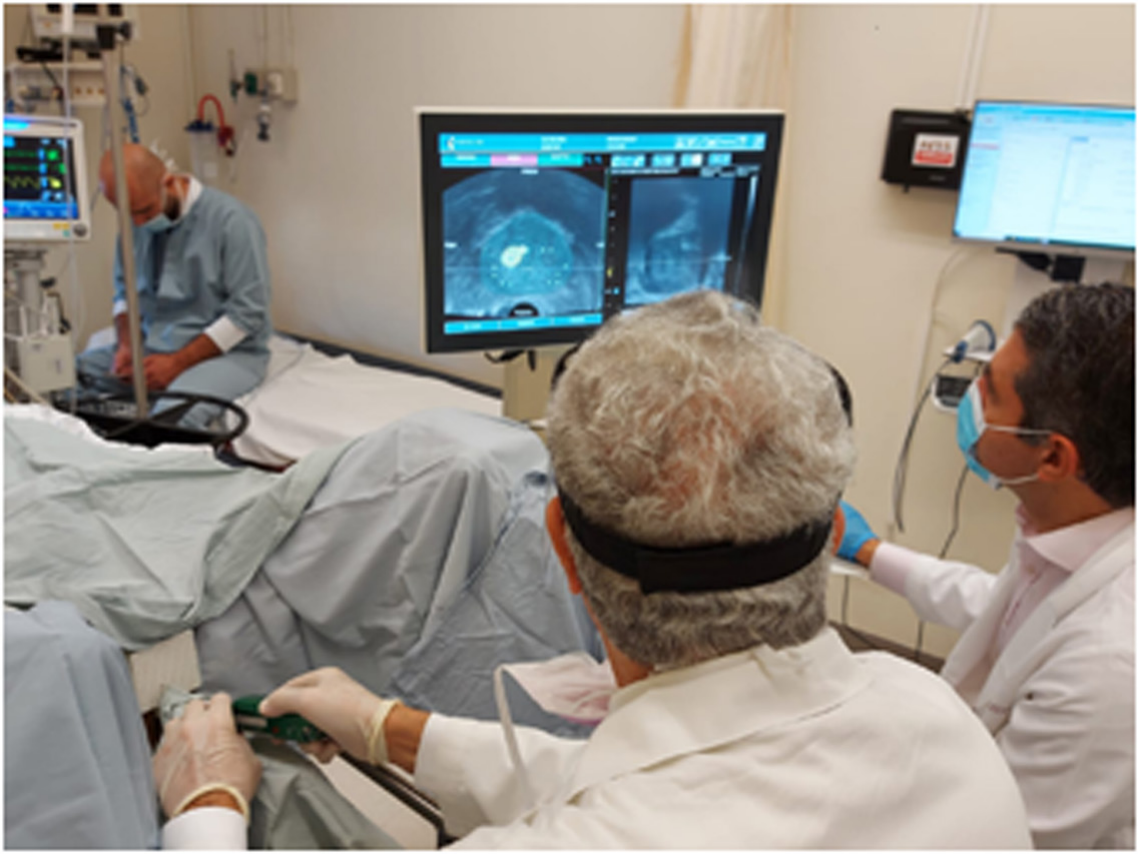
The transperineal biopsy set up: after contouring of the prostate and delineation of lesions on the grid, the urologist and uroradiologist take targeted and random core which are tracked live on display.

After introduction of the TRUS, the prostate edges are contoured and the images of the mpMRI are superimposed on the US images. Suspicious lesions are identified and defined on the 3D image. Targeted cores were fired on suspicious lesion and random cores were fired on uninvolved zones of the prostate. Biopsy cores are tracked live on the US display and saved (Figure 2).

The patient’s pain and discomfort were self-reported after the procedure and were measured using the Wong–Baker Faces pain scale. This pain scale is a series of faces ranging from happy face or “no hurt”at 0 to a crying face at 10 which represents the “worst pain imaginable.” Patients were asked to report their pain scale during the procedure and after it. 

## Results

Between May 2019 and June 2020, 145 patients underwent MRI/US fusion biopsy of prostate at out institution. Forty-seven patients had TRUS biopsy while 98 had TP biopsy. No difference in median age (66 vs. 64.5; *P* = −0.68), median PSA value (7.5 vs. 7.5; *P* = .42), and median prostate volume (51 vs. 52.5; *P* = .83) was reported. More patients in the TRUS group were on 5 alpha reductase inhibitors (25.5% vs. 10.3%; *P* = .025) (Table 1). Three patients (3.2%) were on active surveillance (AS) in TP groups, while only 1 (2.1%) was on AS for TRUS groups. 

All patients had MRI prior to biopsy. A total of 183 lesions were targeted, with similar distribution of PIRADS 3, 4, and 5 between the groups (Table 1). Forty-seven (32%) had 2 or more suspicious lesions on MRI. Fifty-nine lesions were targeted in the TRUS groups and 124 for TP groups. The median (IQR) number of total cores fired were 13 (11-15) versus 11 (9-14) for TRUS versus TP, respectively. Similar number of targeted cores were fired (5 (4-7) vs. 6 (4-7); *P* = .338), but a smaller number of random cores were taken for the TP group (median (IQR) 6 vs. 3; *P* < .0001). There was neither any difference between the detection rate of cancer per patient between TRUS and TP (53.1% vs. 55%; *P* = .83) nor detection rate of CSC per patient (48.9% vs. 44%; *P* = .57). 

**Figure 3.**
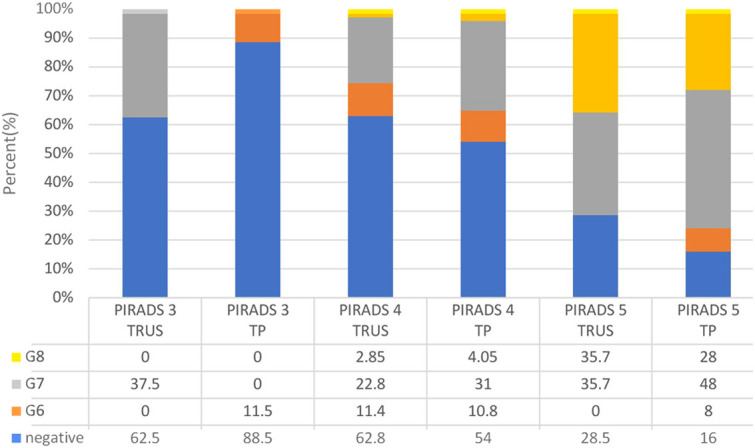
Detection rate of Cancer by Gleason score of Transperineal biopsy versus Transrectal biopsy of prostate. Distribution of Gleason score by PIRADS 3, 4, and 5. TRUS, transrectal biopsy; TP, transperineal biopsy; G, Gleason score.

**Figure 4.**
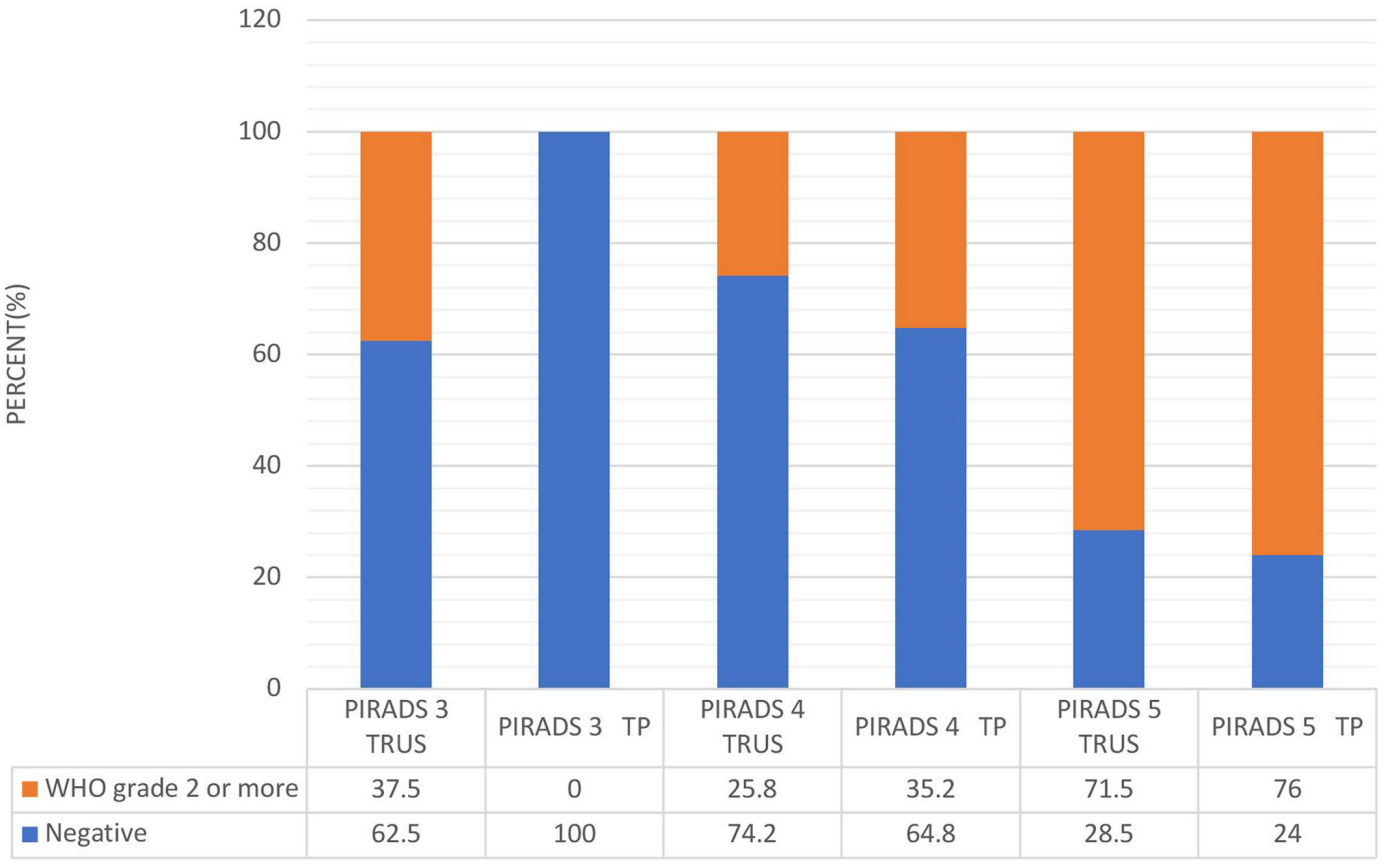
Detection rate of clinically significant cancer (WHO grade 2 or more) by PIRADS in the TP versus TRUS approach. TRUS, transrectal biopsy; TP, transperineal biopsy; G, Sleason score.

The detection rate of CSC in all targeted lesions (n = 183) was similar between TRUS versus TP (40.6% vs. 39.5%; *P* = .69). The distribution of G6, G7, and G8 and above is similar between the groups (Table 2). To note, none of the targeted PIRADS 3 lesions in TP groups showed CSC, while 3 out 9 PIRADS 3 lesions targeted in the TRUS group showed G7 lesions (Figure 3). For PIRADS 4 and 5 lesions, detection rate of CSC was similar between the groups (25.8% vs. 35.2%; *P* = .32, 71.5% vs 76%; *P* = .51), respectively (Figure 4). There was neither any difference in the detection rate of anterior lesion 5 (41%) versus 10 (50 %), *P* = .65 nor in the detection rate of apical lesions 10 (50%) vs 12 (40%), *P* = .49.

Random cores were taken for 43 patients in the TRUS group and 86 in the TP group. More cancer was detected by random cores in the TRUS versus TP (44% vs 15%; *P* = .0001) as well as CSC (35% versus 9.3%; *P* < .0001). However, no statistically significant difference was found between the groups when the target was negative, and the random cores were positive. Three patients in the TRUS group had CSC on random cores, while only 1 in the TP (3 vs. 1; *P* = .09) with a negative target (Table 3). 

To note, none of the TP patients reported any discomfort or pain during or after the procedure. Using the Wong–Baker FACES pain scale, patient had median baseline pain level of 0 and post procedure ranged between 0 and 2. On the other hand, TRUS patients pain scale ranged between 6 and 8, especially during periprostatic block and during the first few cores.

None of the patients in the TP group had retention or hematuria, while in the TRUS group only 1 patient had retention and 1 patient had hematuria significant enough to require irrigation. The rate of sepsis was similar between the groups TRUS versus TP (1 vs. 2; *P* = .2). However, more patient in the TRUS had Lower Urinary tract symptoms (LUTS) and non-culture proven UTI that required antibiotics treatment (5 vs. 1; *P* = .002) (Table 4).

Nineteen patients in TP groups and 13 patients in TRUS group underwent radical prostatectomy with or without lymph node dissection. In TP group, 7 patients upgraded on final pathology compared to the biopsy and 5 downgraded. One patient upstaged and 1 downstaged. On the other hand, in the TRUS group, 3 upgraded, 2 downgraded, 4 patients upstaged, and none downstaged. No significant differences in the rate of upgrading, upstaging post radical Prostatectomy was found between 2 biopsy techniques (Table 5). 

## Discussion

The recent advancements in prostate imaging, specifically prostate mpMRI, has allowed for improvement in the detection of CSC of the prostate with a higher detection rate using standard prostate biopsy templates.^7,15,16^ The importance of MRI in the diagnosis of prostate cancer is underscored in the infamous trials The Prostate MR Imaging Study and PRostate Evaluation for Clinically Important disease: Sampling using Image-guidance Or Not?. Both showed that MRI prior to biopsy decreases the number of unnecessary biopsies and increases the detection of clinically significant prostate cancer.^12,16,17^ Although the 2 main approaches to biopsy are TR and TP, MRI-guided biopsies can be done by many techniques either cognitive, fusion, or in-bore techniques.^18^

This study has shown the non-inferiority of MRI fusion TP prostate biopsy in comparison to MRI fusion TRUS prostate biopsy. Our findings show that during the adoption phase of TP prostate biopsy, there was similar rate of cancer detection (55% vs. 53%) and a non-inferiority in detection of CSC (48.4% vs. 44%) irrespective of Gleason type. Our findings are in accordance with most of the literature.^7,19,20^ A recent systematic review and meta-analysis of studies comparing TP and TRUS MR fusion prostate biopsies showed a similar sensitivity and specificity when comparing the 2 techniques.^21^ Contrarily, another systematic review and meta-analysis of all types of targeted biopsies (not only fusion) comparing the 2 techniques showed an advantage of the TP approach.^22^ Moreover, there are several reports in the literature as well that show the superiority of the TP approach in detection of anterior and transition zone lesions,^18,23,24^ though no difference was found in the detection rate of anterior lesions in our cohort, which is probably due to the small sample size. 

In this study, although TP is non-inferior in terms of detection rate, one can argue that the TP biopsy approach is more accurate. For instance, in this study, there was a similar detection rate of prostate cancer while taking less cores in the TP approach. Less cores mean less complication rates, including UTI, hematuria, and retention.^13,25^ Furthermore, the TRUS biopsy cohort had a higher rate of random biopsy that detected cancer, which could be an indication of a higher accuracy of the TP method, as well as better core quality. In our study similar to Miah et al^26^, there seems to be limited clinical value in adding random cores in the TP prostate biopsy due to the low yield of CSC in random cores in this approach. Future studies are needed comparing the accuracy and quality of cores of TP versus TRUS by correlating prebiopsy MRI, post-biopsy pathology, and location of lesion post-radical prostatectomy.

Regarding patients that had previous negative biopsies, data are still controversial on whether a right 12-core systematic biopsy is needed in addition to targeted core, especially in the current era of mpMRI and fusion biopsy. While some studies have shown that systematic biopsy adds little benefit to the detection rate of CSC compared to fusion in patients who underwent mpMRI prior to repeat biopsy;^27,28^ others have found need to include systematic biopsy in order not to miss 3.4-9% of CSC.^29,30^ Both sides agree that for patients with prior negative biopsy, the fusion biopsy should include some random cores.^31^

Over the last decade and since the first reports of the TP approach for the prostate biopsies,^32^ there has been a growing dispute over the advantages and disadvantages of TP and TRUS approaches. One of the main disadvantages of the TRUS approach is the associated post-procedural infection rate by direct inoculation of rectal bacteria into the prostate.^33^ In contrast to the TRUS approach, the TP route significantly decreases the risk of direct inoculation of bacteria. Grummet et al^34^ pooled more than 6000 men who underwent TP prostate biopsies and showed a sepsis rate of 0.076%. Indeed, in our cohort, we found that the TP biopsy is associated with a significantly lower rate of UTIs requiring antibiotics when compared to the TRUS biopsy. More patients in the TRUS cohort presented with LUTS post biopsy prompting urologist to dispense an extended course of antibiotic prophylactically to patients to avoid sepsis, thus feeding into the never-ending cycle of increased rates of prostatitis with resistant strains of bacteria. In light of all the evidence of non-inferiority of TP prostate biopsy and superiority in decreasing infectious complications, the most recent European association of Urology (EAU) guidelines on prostate cancer detection strongly advocate for the TP approach for prostate biopsy.^35^

One of the main disadvantages of the TP approach mentioned in literature is the higher rate of retention (between 2% and 24%).^20,36,37^ We have found similarly low rates of acute urinary retention between TRUS and TP biopsy, which we believe was due to the lack of periprostatic block and the procedure being done under sedation, as well as the decreased number of cores taken, which are all risk factors for retention status post biopsy.^13,25^

From our experience, TP biopsy performed under light sedation is a much better tolerated procedure than TRUS biopsy of the prostate. In fact, 20% who have had previous TR biopsy, all reported a preference for the TP approach.^38^ Not only was there a further decrease in the motion of the patient’s prostate for better fusion targeting, light sedation was also found to be an excellent form of analgesia for our patients, because they do not experience the pain of the pricks of local anesthesia or periprostatic block.^39^ On the other hand, new, safe, and novel technique has recently been introduced as a substitute to periprostatic block called transcutaneous electrical nerve stimulation. Transcutaneous electrical nerve stimulation is a non-invasive method of controlling pain via activation of endogenous opioid mechanism in the spinal cord through the firing of pulsed electrical currents between 2 electrodes.^40^

This study has few limitations including the retrospective nature of the study. Another one of the limitations is the small sample size, especially the low number of TRUS fusion biopsies. The reason being that with the introduction MRI/US fusion technology at our institution, we started shifting to the TP approach and fewer TRUS biopsies are being done at our center. Lastly, no validated questionnaires were administered to the patient at specific times pre and post biopsy to assess self-reported pain, hematochezia, and hematuria. This is considered a reporting bias. However, we believe we have captured all major complications (infection, antibiotic prescription, retention, and hematuria) requiring medical intervention. 

Comparative data between MRI/US fusion TP versus TRUS showed a better tolerated procedure with similar detection rates of CSC, with a smaller rate of UTI for TP approach. With a fewer number of cores needed for TP biopsy, there are less complications and excellent detection rates of CSC. This is the first and largest series in the Middle East. Transperineal biopsy of the prostate should be the new gold standard to biopsy the prostate. 

## Figures and Tables

**Table 1.** Patient Demographics, Distribution of mpMRI Findings, and Summary of MRI/US-Fusion Biopsy Findings

	TRUS/mpMRI TR Fusion Target Biopsy, N = 47	TRUS/mpMRI TP Fusion Target Biopsy, N = 98	*P* (<.05)
Median age (IQR)	66 (58-72)	64.5 (59-72)	.68
Median PSA (IQR)	7.5(5-13)	7.5(4.8-13.7)	.42
PSA density	0.19	0.22	.5
Median prostate size (IQR)	52.5 (38.5-72)	51 (40-68)	.83
DRE suspicious	57.4%(n = 27)	42.9% (n = 42)	.1
Dominant lesion size (average)	1.25	1.20	.68
Active surveillance (%)	1 (2.1%)	3 (3.2%)	1
Hx of TURP	5 (10.6%)	17 (17.3%)	.29
On 5 alpha reductase	12 (25.5%)	10 (10.3%)	.025
PIRADS (targeted lesions)	59	124	Total [N = 183].8
2	1 (1.6%)	1 (0.8%)	
3	9 (15.2%)	27 (21.7%)	
4	35 (59.3%)	71 (57.2%)	
5	14 (23.7%)	25 (20.1%)	

**Table 2.** Biopsy Results Including Prostate Cancer Detection Rate Stratified by Gleason, Number of Targeted and Random Cores as Well as Detection Rate of Apical and Anterior Lesions

	TRUS	TP	*P*
Median number of cores taken (IQR)	13 (11-15)	11(9-14)	.025
Median target (IQR)	5 (4-7)	6(4-7)	.338
Median random (IQR)	6(4-9)	3(2-6)	<.001
Detection rate of targeted anterior lesions, n (%)	5/12(41.1%)	10/20 (50%)	.65
Detection rate of targeted apical lesions, n (%)	10 (50%)	12 (40%)	.49
Detection rate of prostate cancer, n (%)	25 (53.1%)	54 (55%)	.83
Detection rate of CSC, n(%)	23 (48.9%)	43 (44%)	.57
Detection rate of CSC in all targeted lesions	40.6%	39.5%	.69
G6	4	15	.27
G7	17	32	.65
≥G8	7	17	.73

**Table 3.** Difference of Detection Rates of Cancer and Grade ≥ 2 Cancer on Random Cores Between Transrectal (TRUS) Versus Transperineal Biopsy of Prostate (TP)

	TRUS (N = 45)	TP (N = 98)	*P*
Random biopsy	43 (95.5%)	86 (87.7%)	.14
Positive random	19 (44%)	13 (15%)	.0001
G≥2 grade random	15 (35%)	8 (9.3%)	.0001
Positive CSC random and negative target	3	1	.09

**Table 4.** Complication Rates After Fusion Transperineal (TP) Versus Transrectal Ultrasound Biopsy (TRUS)

	TP	TRUS	*P*
Hematuria	0	1	n/a
Retention	0	1	n/a
Patients with LUTS/UTI prescribed antibiotics	1	5	.006
Sepsis requiring admission (culture proven UTI)	1	2	.2
Septic shock requiring ICU	0	0	n/a

**Table 5.** Rates of Upgrading, Downgrading, Upstaging, and Downstaging in TRUS Versus TP Prostate Biopsy

Robotic or Open Radical Prostatectomy	TRUS (N = 13)	TP (N = 19)	*P*
Upgraded	3	7	.4
Downgraded	2	5	.46
Upstaged	4	1	.05
Downstaged	0	1	n/a
